# Synaptic organic transistors with a vacuum-deposited charge-trapping nanosheet

**DOI:** 10.1038/srep33355

**Published:** 2016-09-20

**Authors:** Chang-Hyun Kim, Sujin Sung, Myung-Han Yoon

**Affiliations:** 1 School of Materials Science and Engineering, Gwangju Institute of Science and Technology, Gwangju 61005, Republic of Korea; 2 Research Institute for Solar and Sustainable Energies, Gwangju Institute of Science and Technology, Gwangju 61005, Republic of Korea

## Abstract

Organic neuromorphic devices hold great promise for unconventional signal processing and efficient human-machine interfaces. Herein, we propose novel synaptic organic transistors devised to overcome the traditional trade-off between channel conductance and memory performance. A vacuum-processed, nanoscale metallic interlayer provides an ultra-flat surface for a high-mobility molecular film as well as a desirable degree of charge trapping, allowing for low-temperature fabrication of uniform device arrays on plastic. The device architecture is implemented by widely available electronic materials in combination with conventional deposition methods. Therefore, our results are expected to generate broader interests in incorporation of organic electronics into large-area neuromorphic systems, with potential in gate-addressable complex logic circuits and transparent multifunctional interfaces receiving direct optical and cellular stimulation.

Today, organic electronics stands at a crossroads of development. The early demonstration of the optoelectronic functionalities of molecular/polymeric materials has generated massive research outcomes over the last few decades^1^,^2^,^3^,^4^,^5^, whereas industrial acceptance still seems to be slow due to the difficulty in finding applications that truly maximise the unique potential of organic semiconductors^6^,^7^,^8^. Thin-film transistors, light-emitting diodes, and photovoltaics have constituted the first generation of organic optoelectronics families, and the quest for novel electronic systems with unusual functions is now gaining more attention. In this context, neuromorphic systems that mimic functionalities of biological neurons still remain largely unexplored within the organic electronics community. The stated goal of traditional neuromorphic circuits relying on inorganic materials is to overcome the von Neumann bottleneck and to construct a brain-like computer that performs complex data processing and stores massive information in a faster and more energy-efficient way^9^. Although an organic-based neuromorphic network may not be intended to compete with its inorganic counterpart in terms of processing speed and memory capacity, organic semiconductors’ solution processability, mechanical flexibility, and biocompatibility are judged to be promising for large-area applications as well as direct bio-interfacing hybrid systems.

An electronic synapse is an essential element for neuromorphic systems^10^, and an organic semiconductor-based synaptic transistor (synapstor) was pioneered by Vuillaume and co-workers^11^,^12^. In this device architecture, the short-term retention of charge carriers within a memory transistor channel was utilised to emulate activity-dependent response at the biological synaptic junction between two neurons^13^,^14^,^15^. Although the working principles of organic synapstors were successfully demonstrated in their seminal work using a hybrid of solution-deposited Au nanoparticles and a vapour-processed pentacene film, the nanostructured morphology at the dielectric-semiconductor interface resulted in a disordered semiconductor layer, thereby causing an inevitable trade-off between charge transport and memory performance^11^,^16^,^17^. Also, additional elaboration of molecular self-assembly on nanoparticle surface was required for charge barrier and for good adhesion to the substrate, while performance variation could be caused by moderate surface anchoring and random distribution of the particles^16^.

In this article, we report on a new synapstor concept that is devised to fabricate organic-based neuromorphic devices with a high channel conductance and an activity-dependent memory characteristic. A vacuum-deposited metallic nanosheet provides an ultra-flat semiconductor-dielectric interface optimum for the growth of an organic channel, and offers a decent but temporary charge-immobilisation capability. Accompanying detailed structural and electrical characterisations suggest that this simple, reliable, and highly scalable implementation, based on widely available materials and manufacturing protocols, can be a promising route toward developing advanced memory circuits and functional neuro-inspired electronics.

As depicted in Fig. 1a, the organic field-effect transistor (OFET)-based synaptic devices were constructed on top of a flexible and transparent polyethylene terephthalate (PET) substrate, with a stack made of indium-tin-oxide (ITO)/poly(3,4-ethylenedioxythiophene):poly(styrenesulfonate) (PEDOT:PSS)/poly(methyl methacrylate) (PMMA), which serves as a common gate electrode/dielectric platform^18^. A thin PEDOT:PSS buffer layer reduces the roughness of ITO surface^19^, and therefore utilising PEDOT:PSS was effective in mitigating gate leakage current (*I*_*G*_) and associated device failures (Supplementary Fig. S1). Among the important requirements for synaptic operation is the volatile charge storage, which allows for the fast modulation of charge density upon driving. In this work, an ultra-thin metallic film, with a tunnelling layer formed via ambient oxidation, was incorporated into the transistor structure for the purpose of short-term charge retention. Briefly, an Al nanosheet (3 nm) was deposited via thermal evaporation and exposed to the ambient air for native oxide formation^20^. The intact metallic part between PMMA and aluminium oxide thus appears analogous to the conventional floating-gate electrode^21^. Subsequently, a stable high-mobility organic semiconductor dinaphtho[2,3-b:2′,3′-f]thieno[3,2-b]thiophene (DNTT)^22^ was vacuum-deposited directly on top of this self-formed floating gate (SFG), followed by the evaporation of Au source/drain top contacts through a shadow mask. The atomic force microscopy (AFM) images of the DNTT films show that molecular grains are well defined with clear interlayer terraces, which should be favourable for in-plane charge-carrier transport (Fig. 1c)^23^. Considering that the root-mean square roughness of the Al-SFG-coated PMMA surface (0.19 nm) was similar to that of bare PMMA (0.16 nm), the channel performance will not seriously deteriorate by the incorporation of this memory interlayer^24^. As confirmed by the AFM topography and cross-sectional transmission electron microscope (TEM) images (Fig. 1c,d and Supplementary Fig. S2), we obtained a continuous and smooth Al sheet, in contrast to the previous reports where a-few-nm thin evaporated Al often appeared as nanoparticles or islands^25^,^26^. Two-dimensional (2-D) energy-dispersive X-ray spectroscopy (EDS), conducted on the cross-section of the Al SFG, also showed the formation of a continuous Al band along the PMMA surface in conjunction with a direct proof of oxidation (Fig. 1e). Note that a considerably slow thermal evaporation (with a rate generally lower than 0.05 nm/s) of Al under a high vacuum condition was the key to achieve a continuous, ultra-smooth nanosheet in a reproducible manner. Indeed, we found that a deposition of Al on PMMA with an elevated rate of 0.1 nm/s produces particle-like domains for the same nominal thickness (Supplementary Fig. S3). On the other hand, the final film structure was less sensitive to other experimental conditions as long as the underlying insulator surface is flat.

Figure 2a shows the transfer curves [drain current (*I*_*D*_) versus gate voltage (*V*_*G*_)] of OFETs with and without a SFG, in which the static electrical bias was applied to understand the effect of SFG incorporation on the basic device characteristics. The transistor with an Al SFG exhibited an enlarged hysteresis window, while its current on-off ratio is comparable to that without an Al SFG. A similar trend of enlarged hysteresis without sacrificing the transistor on-off ratio was also observed in OFETs made of pentacene in combination with a PMMA or SiO_2_ dielectric layer (Supplementary Fig. S4). While these results clearly extend the applicability of our concept, pentacene-based devices generally showed lower performance; therefore, we focus on the detailed characterisations using the DNTT devices hereinafter. It is reasonable to speculate that, while the thickness of Al_2_O_3_ is not directly determined in this work, part of metallic Al exists as non-oxidised, because a fully oxidised Al layer would not function as a charge-trapping memory centre^20^. As shown in Fig. 2b, the output characteristics [*I*_*D*_ versus drain voltage (*V*_*D*_)] with clear linear-to-saturation transition confirm the proper channel pinch-off and minimal contact resistance^27^,^28^. Additionally, the dual-sweep output curves of a SFG-OFET show the characteristic memristive behaviour that originates from the charge trapping and release taking place within the timescale of curve sweeps^29^. We obtained a near-100% yield of working devices^30^, benefiting from our geometrically flat, and spatially uniform 2-D memory element (Fig. 1d). All of the 36 devices realised on a 1.5 cm × 1.5 cm PET substrate were operational with very low fluctuations in their performance. Figure 2c shows on-chip statistics of field-effect mobility (*μ*) extracted from SFG transistors, obtained by estimating the slope of the square-root of the hysteresis-averaged saturation-regime *I*_*D*_^31^,^32^. This distribution corresponds to *μ* of 0.94 ± 0.23 cm^2^V^−1^s^−1^, averaged over the full set of 36 FETs. Figure 2d shows that charge-transport and switching capabilities of the SFG-OFETs, parameterised by *μ* and the transconductance (*g*_*m*_ = *dI*_*D*_/*dV*_*G*_), are comparable to or even slightly better than those of the PMMA-only OFETs. This clearly indicates that the quality of a molecular channel in our nanosheet-based transistors is promising, as compared to OFETs with nanoparticles that showed the apparent reduction of *μ* by two to three orders of magnitude^11^. It was also found that the film microstructure as well as electrical conduction of DNTT is less sensitive to the existence of an Al SFG than that of pentacene. The *μ* of pentacene OFETs decreased slightly with an inclusion of an Al SFG, from 0.25 cm^2^V^−1^s^−1^ to 0.14 cm^2^V^−1^s^−1^. This observation can be attributed to the mitigated dendritic growth of pentacene grains on the Al SFG (Supplementary Fig. S5). The energy diagram in Fig. 2e illustrates mechanism of the charge-trapping based memory. Considering the negligible density of thermal carriers in a vacuum-processed organic semiconductor and the large electron-injection barrier from Au to DNTT, hole injection from the source electrode dominates the device current^33^. When a strong gate field is applied at a negative *V*_*G*_, part of the accumulated holes are injected into the Al region due to the tunnelling transparency of the thin oxide, and it results in the decrease in the number of free carriers during the reverse gate sweep. A gate-charging experiment in Fig. 2f also supports this mechanism. After the Al-SFG containing device is charged with a constant *V*_*G*_ of −20 V for 10 s (with *V*_*D*_ = 0 V), there is a considerable shift of the transfer characteristic in the negative-*V*_*G*_ direction, while its slope remained practically unchanged. This manifests that the memory effect is mainly electrostatic with the channel property practically unaffected, and the observed threshold voltage shift is explained by the temporal immobilisation of holes within the SFG. We additionally verified the stability of our SFG-OFETs during repeated electrical addressing and continuous operation. Upon application of multiple current-voltage sweeps, the channel current gradually decreased evidencing the prompt action of the charge-trapping nanosheet; however, the FET recovered its initial current level after a certain period of relaxation (Supplementary Fig. S6). A bending-flexibility test was also carried out, and stable electrical operation was observed under a moderate stress condition (Supplementary Fig. S7).

The static current-voltage characteristics of OFETs reflect the combined effect of carrier accumulation/depletion dynamics and their drift along the channel. Therefore, small-signal impedance analysis was performed to systematically decouple these two contributions and quantitatively analyse induced and trapped charges. Schematics of the two types of device structures studied are shown in Fig. 3a. First, to verify the basic dielectric property of a PMMA film in the configuration of metal-insulator-metal (MIM, Fig. 3a top), its total complex impedance (*Z*_MIM_) was recorded as the vector ratio between applied ac phasor voltage (*V*_PH_) and measured phasor current (*I*_PH_)^34^. The active area (*A*) of the MIM diode and the thickness of the PMMA layer were 0.09 cm^2^ and 410 nm, respectively. The Bode plot in Fig. 3b indicates that our MIM device practically follows the characteristic frequency (*f*) response of a series connected resistance (*R*)-capacitance (*C*) circuit. This reflects high-quality electrical insulation of the PMMA film with minimal leakage. The series *R* may account for the conduction through the PEDOT:PSS buffer and/or other possible resistive loss in the course of contacts and wiring. By modelling the measured impedance with a series *R*-*C* equivalent circuit, we obtained *R* = 430 Ω, *C* = 700 pF (*C*/*A* = 7.7 nF/cm^2^). Next, the gate-controlled charge accumulation/depletion behaviour of the OFET devices was evaluated in the configuration of pseudo metal-insulator-semiconductor (MIS) as shown in Fig. 3a (bottom). Capacitance was measured at a fixed *f* while a dc voltage sweep was applied in the subsequent off-to-on and on-to-off scan directions (Supplementary Fig. S8). In Fig. 3c, the area under the *C*-*V*_*G*_ curve is equivalent to the net change of gate-induced charges with respect to the starting point of the voltage sweep^35^,^36^. Therefore, the accumulated (positive) charge *Q*_acc_ and the depleted charge *Q*_dep_ were obtained by numerically integrating the *C*-*V*_G_ curves with the *V*_*G*_ scan from +10 to −10 V and from −10 to +10 V, respectively. The trapped charge (*Q*_tr_) after a bi-directional sweep was calculated using the relationship *Q*_tr_ = *Q*_acc_ − *Q*_dep_; all *Q*’s were then normalised by using the effective accumulation/depletion area. The results in Fig. 3d indicate that the hole-trapping capability in the SFG device is much more pronounced than that in the PMMA-only device. The decrease in *Q*_tr_ with increasing *f* can be associated with *f*-dependent charge-trapping/releasing efficiencies of the Al floating-gate electrode.

Delicate charge modulation through an ultrathin tunnelling oxide and the high-quality carrier transport in a DNTT channel make our platform suitable for neuromorphic data processing, particularly, for mimicking the short-term plasticity (STP) of a biological synapse^37^. Note that such an STP behaviour of biological synapses is accounted for by various dynamic processes and that it forms the basis for the models of higher-order brain functions, including learning, memory, logic, etc^14^,^15^. Before analysing the STP characteristics of our devices, we examined basic drain-pulse responses with *V*_*G*_ of different polarities being applied. As shown in Supplementary Fig. S9, we observed decreasing or increasing magnitude of *I*_*D*_ by a negative or positive *V*_*G*_, with constant *V*_*D*_ spikes. This simple implementation demonstrates that the effective control over floating-gate charge density by an external gate bias can be combined with the electrical conduction of the channel to achieve programmable current modulation. Figure 4a shows the driving scheme for pulsed activations, where the shape of input spike train conditions the intensity of output spikes^11^. The responses to synaptic input pulses (*V*_syn_) in the SFG OFETs are displayed in Fig. 4a (bottom). Here, two representative devices from the same substrate are labelled as synapstor 1 and 2, and their electrical characteristics are shown together to demonstrate the performance uniformity. Noticeably, effective modulation of output current (*I*_syn_), in either a decreasing (depression) or an increasing (potentiation) trend, was successfully achieved by simply changing the input spike interval at a constant bias. To make an analogy with a biological synapse, the pre- and post-synaptic neurons are also drawn in Fig. 4a. A possible mechanism for the observed STP is suggested in the following. The strength of the electrical signal at the receiver part (post-synaptic or output load of the transistor) proportionally correlates with the number of free carriers available at the moment of input activation. Note that the pre-synaptic pulse in our configuration serves a dual function of delivering a *V*_*G*_ signal for charge trapping and a *V*_*D*_ signal for current driving. More specifically, a negative *V*_syn_ transfers holes from the DNTT channel into the floating gate while the transport of free holes is simultaneously triggered along the channel. This process emulates the release of neurotransmitters in a biological synapse in response to the spiking pre-synaptic neuron. A compensating mechanism is the natural recovery of charge carriers in the channel via back-tunnelling of trapped holes through the ultra-thin oxide in the SFG structure, which mimics the re-uptake of neurotransmitters at the synaptic cleft of a real neuron. Therefore, the plastic behaviour is dictated by the frequency of input signal relative to the rate of carrier recovery; the ‘depression’ behaviour appears if the number of free carriers gradually diminishes due to the high-frequency spike while the ‘potentiation’ behaviour prevails when they substantially rise in number due to the low-frequency one.

It is inferred that various systems involving temporal/spatial charge motions can lead to similar STP behaviours. For instance, slow polarisation of ferroelectric segments and/or ionic conductance within the gate insulator can presumably replace a floating gate structure, while trapping components such as nanoparticles integrated into the channel might show similar responses. In comparison to these cases, our concept assigns the charge-modulation and transport properties to the physically separate parts of the device, and tunability can be greater than the polarisation-based mechanisms where the material’s response time would limit the transit speed. The transient behaviours are supposed to be dominated by both the retention time constant of the SFG, and the amount of charges stored in the SFG at the moment of activation. As shown in Fig. 4a (bottom), the sharp drop of the magnitude of *I*_syn_ at the first stage sets the floating-gate electrode at the strongly (and positively) charged regime. At the later stages, we observe that a transition between depression and potentiation regimes occurs at roughly between 1 and 2 Hz. This directly translates into the time scale of hole retention of the SFG, which in this case is 0.5 to 1 s. The susceptibility of synaptic function was quantified by the signal modulation factor, defined as the ratio between *I*_syn_ values at the starting and ending points of each constant-frequency stage. The results in Fig. 4b show that, in addition to the substantially larger overall swing range of *I*_syn_ of the SFG device, the per-stage comparison shows its more dynamic communication during both the depression and potentiation periods.

Considering the materials and device dimensions, our synapstors operate at a relatively low driving voltage, and this was enabled by the high-mobility DNTT channel and efficient charge modulation via SFG. Note that an input voltage exceeding 10 V has often been utilised for the demonstration of neuromorphic transistors^11^,^38^,^39^. Figure 4c shows the modulation of the magnitude of *I*_syn_ in our devices by varying the magnitude of *V*_syn_. This plot was obtained by taking the mean of the *I*_syn_ values upon receiving an input spike train of the same frequency sequence as in Fig. 1a, averaged over 5 devices. This result manifests orders-of-magnitude enhancement of *I*_syn_ by only a several-volt change in the applied *V*_syn_, and it shows that the operation voltage can be optimally tuned to power a specific output load. Additionally, the inset of Fig. 4c further demonstrates that the relationship follows a quasi-linear trend, thus allowing for systematically choosing a proper value. We nevertheless acknowledge that, for the organic synapstors to potentially interface with biological cells, a further drop in operation voltages would be necessary, and this would be enabled by using a thin high-*k* dielectric, or by employing an electrolytic gate capacitor^35^.

There are several advantages and drawbacks of three-terminal neuromorphic devices based on the transistor architecture as compared to two-terminal ones based on the resistive-memory structure^39^,^40^,^41^. Despite structural complexity, transistors provide an additional driving option through a gate potential, and can be more operationally stable than memristors that typically involve structural changes within the active material^41^. Note that our neuromorphic transistors can therefore offer the advantage of reliable operation and versatile addressing, while they also benefit from the self-formed electrode/barrier structure that substantially simplifies the manufacturing by not including any intentional deposition of a tunnelling dielectric. Other three-terminal neuromorphic devices reported in the literature include silicon transistors with a charge-storage electrode^39^, indium-zinc-oxide transistors with proton-conducting dielectric^42^, PEDOT:PSS electrochemical transistors^43^ and ionic-liquid-gated samarium nickelate transistors^44^. Each of these systems, including our SFG-based organic synapstor, holds its own distinctive merits in terms of processability, switching performance, response speed, memory capacity, and compatibility with the liquid or solid environment.

In conclusion, we have developed a synapstor structure with a high channel conductance and an efficient memory characteristic using a metallic nanosheet and organic materials. One of the foremost findings was that the distributed capacitive elements such as metallic nanoparticles were not a requisite for volatile charge-based memory, and the planar SFG architecture demonstrated its unique advantages in terms of ease of fabrication, reliability in operation, and excellence in performance. The present study has focused mainly on the quantitative analyses and device-level characterisation, and therefore further investigation will be directed toward the inter-device communications and higher-level neuromorphic functions. Our devices will serve as a promising building block for constructing flexible neuron-inspired circuits, and for demonstrating unconventional bio-interfaces with preferred mechanical and optical properties.

## Methods

### Device fabrication

**Figure 1:**
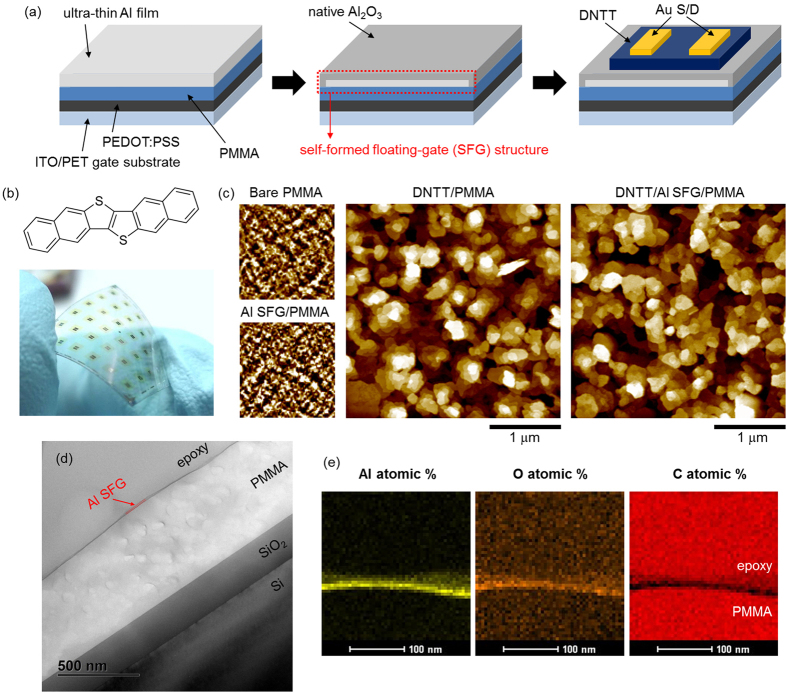
Structural characteristics of the self-formed floating-gate (SFG) architecture. (**a**) A schematic representation of flexible synapstors based on the SFG concept. (**b**) Chemical structure of DNTT and a photograph of flexible OFET arrays. (**c**) AFM topography images. Scan size is 1.0 μm × 1.0 μm for the ‘Bare PMMA’, ‘Al SFG/PMMA’ images and 3.0 μm × 3.0 μm for the ‘DNTT/PMMA’, ‘DNTT/Al SFG/PMMA’ images. (**d**) Long-range cross-sectional TEM image showing the formation of an ultra-thin, continuous Al SFG layer on PMMA. (**e**) 2-D EDS data representing the elemental compositions of Al (yellow: left), O (orange: middle), and C (red: right) at the interface between SFG and PMMA.

The OFETs were fabricated with the bottom-gate top-contact configuration depicted in Fig. 1a. ITO-coated PET substrates (surface resistivity: 60 Ω/sq, Sigma-Aldrich) were rinsed with acetone and isopropanol and dried with nitrogen blow before use. A PEDOT:PSS (Clevios™, Heraeus) buffer layer (40 nm) was spin-coated at 3000 rpm for 60 s, and annealed at 100 °C for 10 min. PMMA (M.W. = 120,000, Sigma-Aldrich) was dissolved in toluene at a concentration of 60 mg/mL and spin-coated at 2000 rpm for 45 s, with annealing at 120 °C for 1 hr, for a 410-nm thick dielectric film. For SFGs, 3 nm of Al was vacuum-evaporated (base pressure: 3.6 × 10^−6^ Torr, rate: 0.01 nm/s). The substrates were exposed to the ambient air for 30 min before being introduced to a vacuum chamber for semiconductor deposition. No additional process such as a prolonged exposure to the air or an oxygen-plasma treatment was necessary for assuring the full oxidation of the Al surface. For both PMMA-only and Al-SFG devices, DNTT (sublimed grade, 99%, Sigma-Aldrich) was thermally evaporated to form a 50-nm thick hole-transporting organic channel (base pressure: 3 × 10^−6^ Torr, rate: 0.02 nm/s). Au source/drain electrodes (30 nm) were finally formed by thermal evaporation through a shadow mask, with a channel width and length of 500 μm and 50 μm, respectively (base pressure: 3.5 × 10^−6^ Torr, rate: 0.03 nm/s). For the MIM capacitor, the substrate cleaning, and formation of the PEDOT:PSS/PMMA layers were conducted using the same process parameters as those used for the OFETs. The top Al contact (50 nm) was vacuum-evaporated through a shadow mask (base pressure: 3.5 × 10^−6^ Torr, rate: 0.1 nm/s).

### Structural characterisation

The surface morphology of the DNTT, pentacene, PMMA, and Al films was investigated by using AFM (XE-Bio, Park Systems). Cross-sectional TEM and 2-D EDS data were obtained by using the TECNAI F30 Super-twin G^2^ model.

### Electrical characterisation

The current-voltage characteristics of OFETs were measured by using the Keithley 4200 Semiconductor Characterization System in the dark under ambient atmosphere. The small-signal electrical impedance was measured by using an integrated capacitance-voltage measurement unit (Model 4200-CVU) in the Keithley 4200 equipment, with the ac voltage of the magnitude of 50 mV. Synaptic characteristics were measured by using a LabVIEW-programmed Keithley 2400 Source Measure Unit.

## Additional Information

**How to cite this article**: Kim, C.-H. *et al*. Synaptic organic transistors with a vacuum-deposited charge-trapping nanosheet. *Sci. Rep.* **6**, 33355; doi: 10.1038/srep33355 (2016).

## Supplementary Material

Supplementary Information

## Figures and Tables

**Figure 2:**
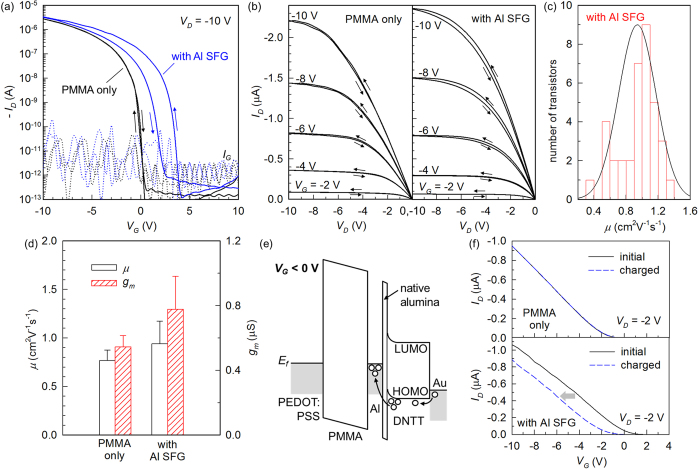
Static electrical characteristics of the OFET devices. (**a**) Transfer and (**b**) output hysteresis curves of the DNTT transistors with or without the SFG structure. The arrows indicate the voltage-sweep direction. (**c**) Statistical distribution of the *μ* values extracted from the DNTT SFG-OFET arrays (36 devices) on a single substrate. (**d**) Statistical comparison of mobility (*μ*) and transconductance (*g*_*m*_ at *V*_*G*_ = −10 V) from PMMA-only and Al-SFG devices. Vertical bars indicate mean values while error bars correspond to standard deviations out of 36 FETs. (**e**) Energy diagram illustrating the proposed mechanism of charge-carrier trapping in the floating gate (*E*_*f*_: Fermi level of the electrodes). (**f**) Transfer characteristics before and after a negative-*V*_*G*_ charging.

**Figure 3:**
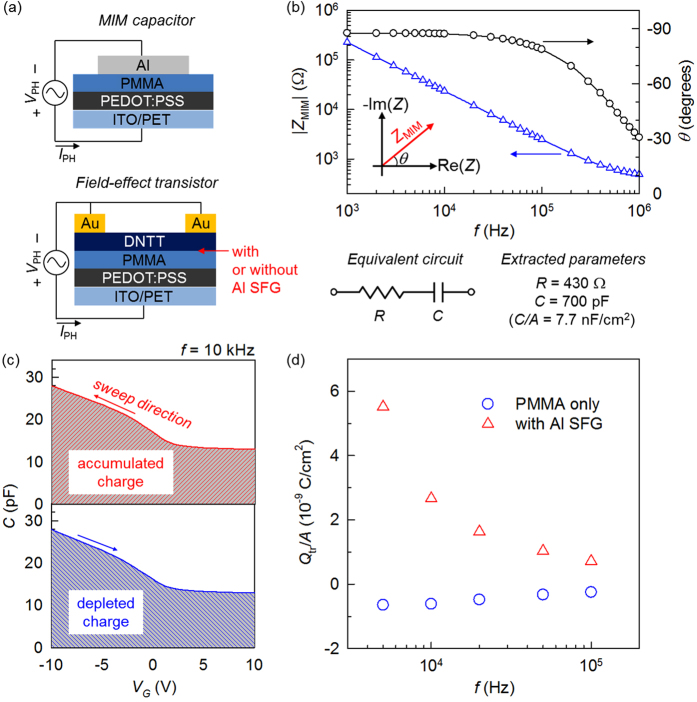
Small-signal impedance analysis. (**a**) Device configurations with the wiring scheme. (**b**) Bode plot for the impedance of the MIM diode (inset: complex plane illustrating the vector *Z*_MIM_ and its phase angle *θ*). Below the plot, the equivalent circuit and the corresponding parameters are presented. Impedance was measured with no dc bias. (**c**) Dual-sweep *C*-*V*_*G*_ curves for the estimation of the gate-induced/depleted charge density in the DNTT SFG-OFET. (**d**) Trapped charge density as a function of small-signal frequency *f*.

**Figure 4:**
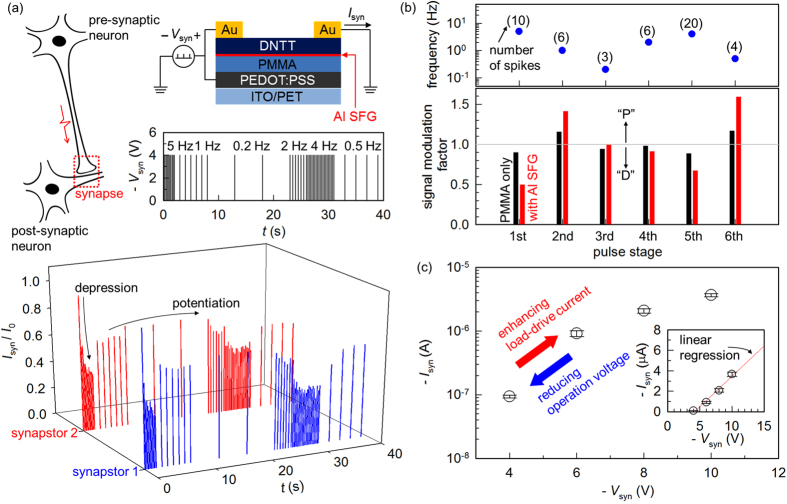
Synaptic operation of the SFG-OFETs. (**a**) Illustration of the neuronal signal transmission through a synaptic connection, neuromorphic device scheme using our DNTT SFG-OFET, time-domain input synaptic voltage (*V*_syn_) pulses, and the measured synaptic currents (*I*_syn_) from representative devices. Measured currents were normalised by the initial value *I*_0_. (**b**) Per-stage signal amplification/attenuation factor calculated for devices with (red) and without (black) an SFG layer. “P” and “D” denote ‘potentiation’ and ‘depression’, respectively. (**c**) The quantitative correlation between the magnitudes of *V*_syn_ and *I*_syn_ in a logarithmic scale. The inset represents the same data in a linear scale, with a linear regression line.
