# Supplementary material for: Synaptic organic transistors with a vacuum-deposited charge-trapping nanosheet
Source: Sci Rep. 2016 Sep 20;6:33355. doi: 10.1038/srep33355 (PMC5028755; doi:10.1038/srep33355)
Supplement: Supplementary Information [file srep33355-s1.pdf]

Supplementary Information

Synaptic organic transistors with a vacuum-deposited charge-trapping nanosheet

Chang-Hyun Kim, Sujin Sung and Myung-Han Yoon*

*E-mail: mhyoon@gist.ac.kr

This “Supplementary Information” includes:

Figures S1 to S9

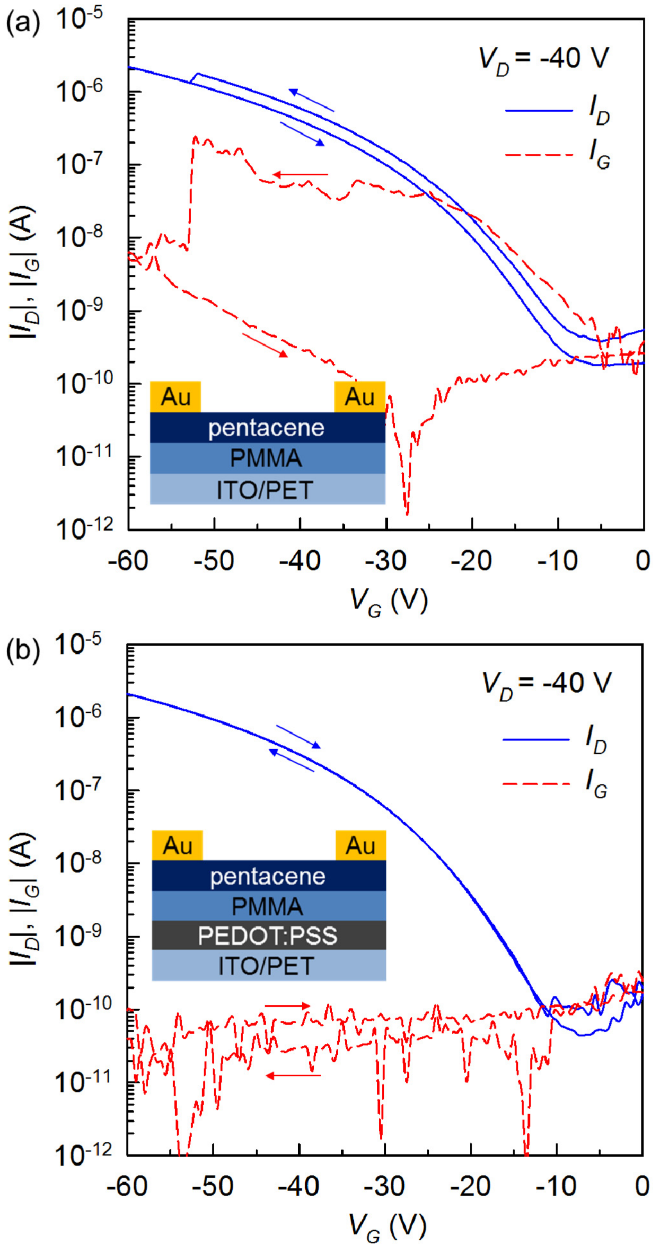

Figure S1. Transfer characteristics of OFETs (a) without and (b) with a PEDOT:PSS buffer layer residing between PMMA and ITO/PET substrate. Device structures are illustrated in insets.

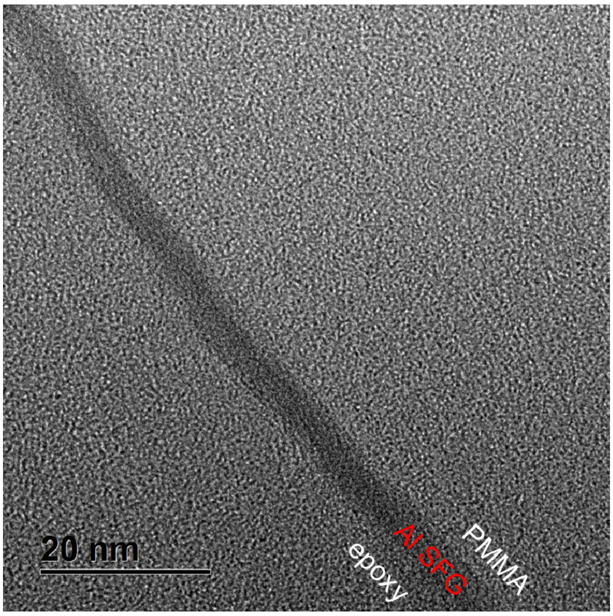

Figure S2. Short-range TEM image showing a conformable growth of an Al nanosheet on PMMA.

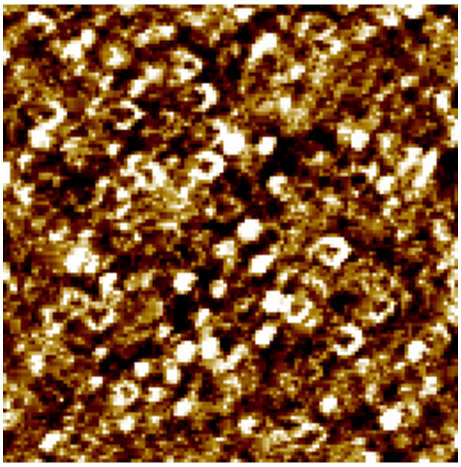

Figure S3. AFM image on the 3 nm of Al deposited on PMMA with an evaporation rate of 0.1 nm/s. Scan size is 1.0 μm × 1.0 μm.

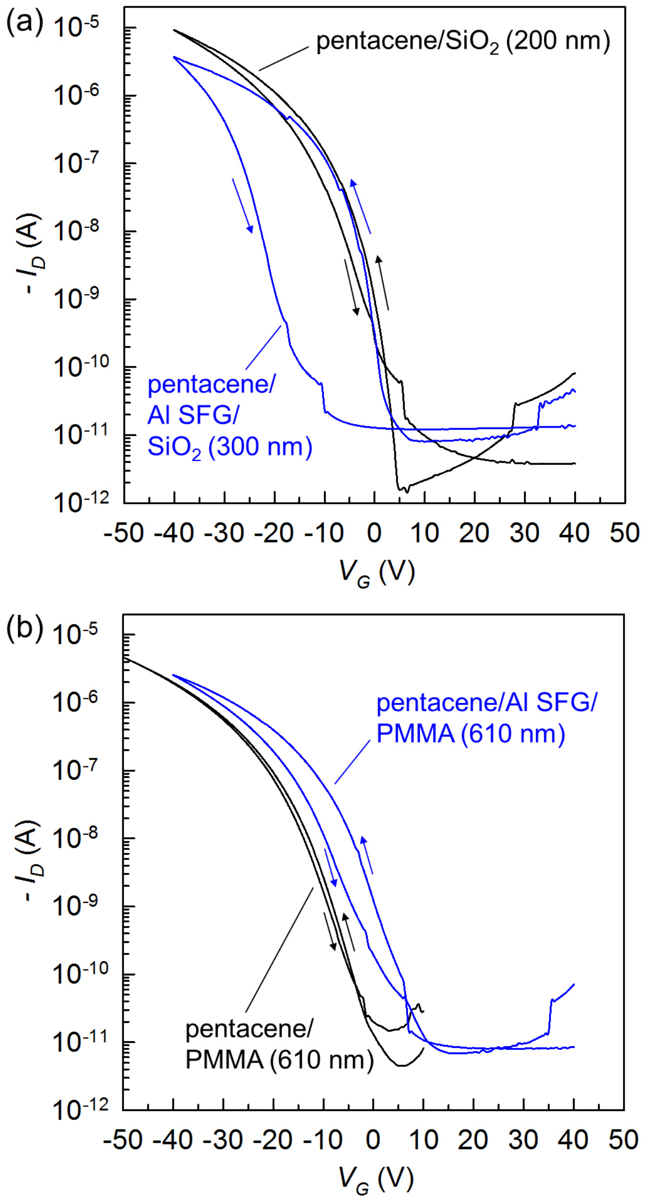

Figure S4. Dual-sweep transfer characteristics measured on pentacene devices with (a) heavily doped Si gate and SiO_2 dielectric and (b) PEDOT:PSS/ITO/PET gate and PMMA dielectric. All devices have a bottom-gate top-contact structure with a 50 nm pentacene channel and Au source/drain contacts. The applied V_D is -50 V for the pentacene/PMMA curve and -40 V for the other three curves. The arrows indicate the direction of voltage sweep.

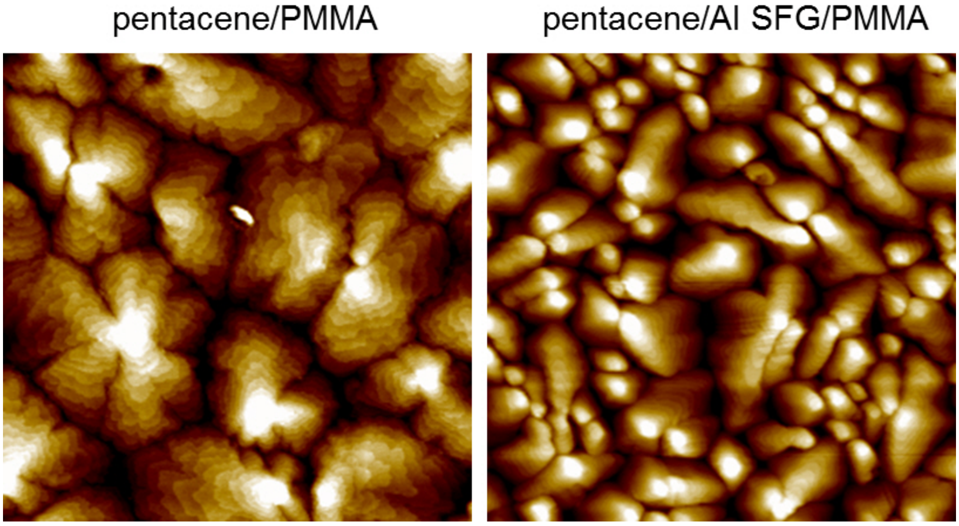

Figure S5. AFM topography images on the pentacene thin film (50 nm) evaporated on the bare PMMA surface (left) and on the Al SFG coated PMMA surface (right). Scan size is 3.0 μm × 3.0 μm for both images.

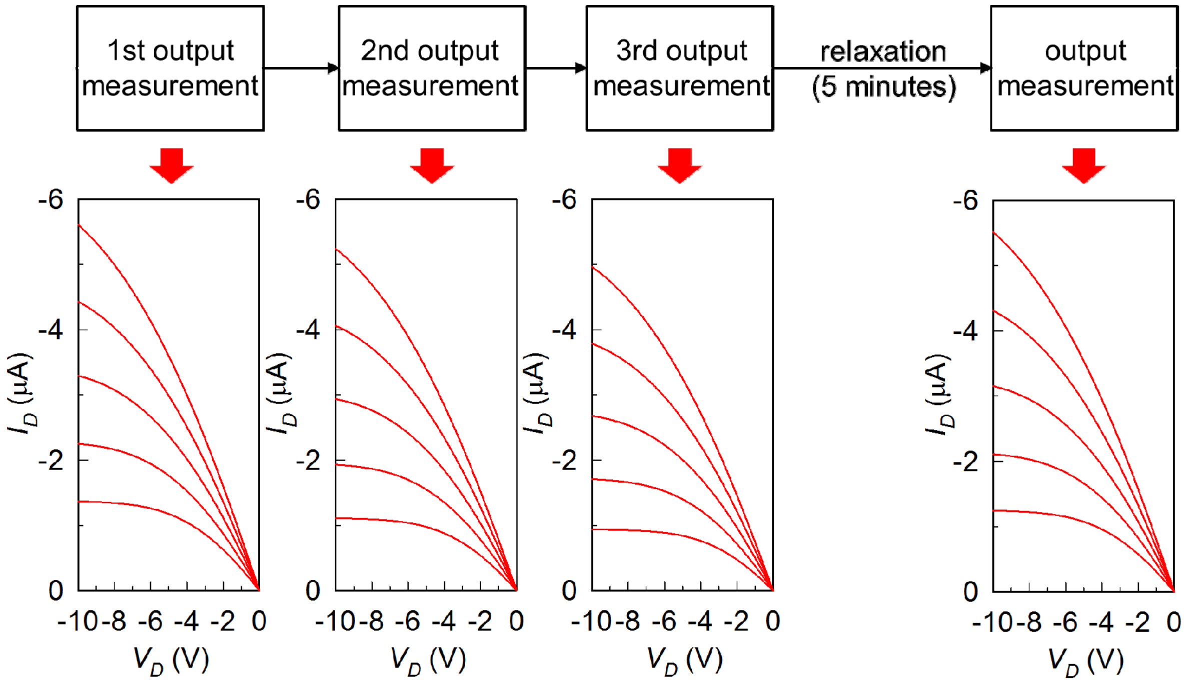

Figure S6. Measurement scheme for the bias effect and relaxation of the DNTT Al-SFG FET, and the corresponding output characteristics (measured at V_G = -2, -4, -6, -8, -10 V). Three consecutive output measurements were conducted with no delay in-between, and after the third measurement, the device was left un-biased for 5 minutes before the final measurement. The series of output data here shows that the drain current gradually decreased during the repeated measurements due to the charge trapping by the floating gate, however, the device recovered its initial state after the trapped charges were released back to the channel.

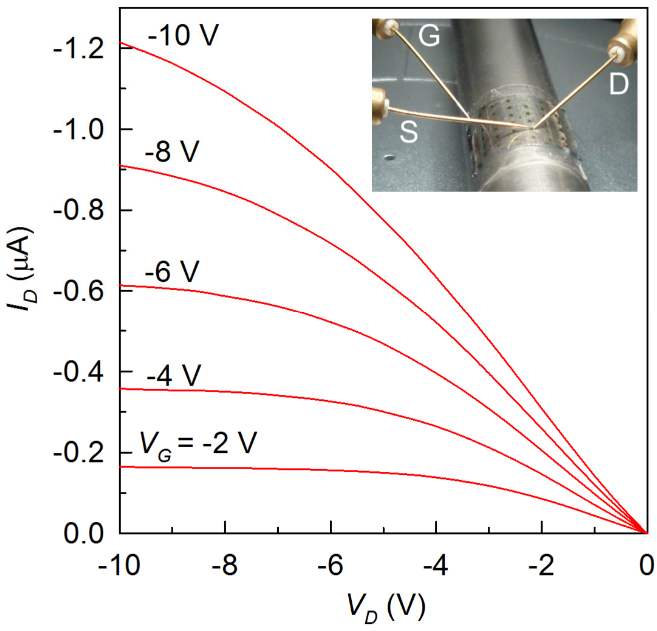

Figure S7. Output characteristics of a DNTT channel Al-SFG FET device under a bending flexibility test, for which the sample was conformably attached to a steel cylinder (inset). The substrate was aligned with respect to the curved surface in such a way that the stress is applied along the direction of the charge-carrier transport (source-to-drain direction). The bending radius is 7.5 mm.

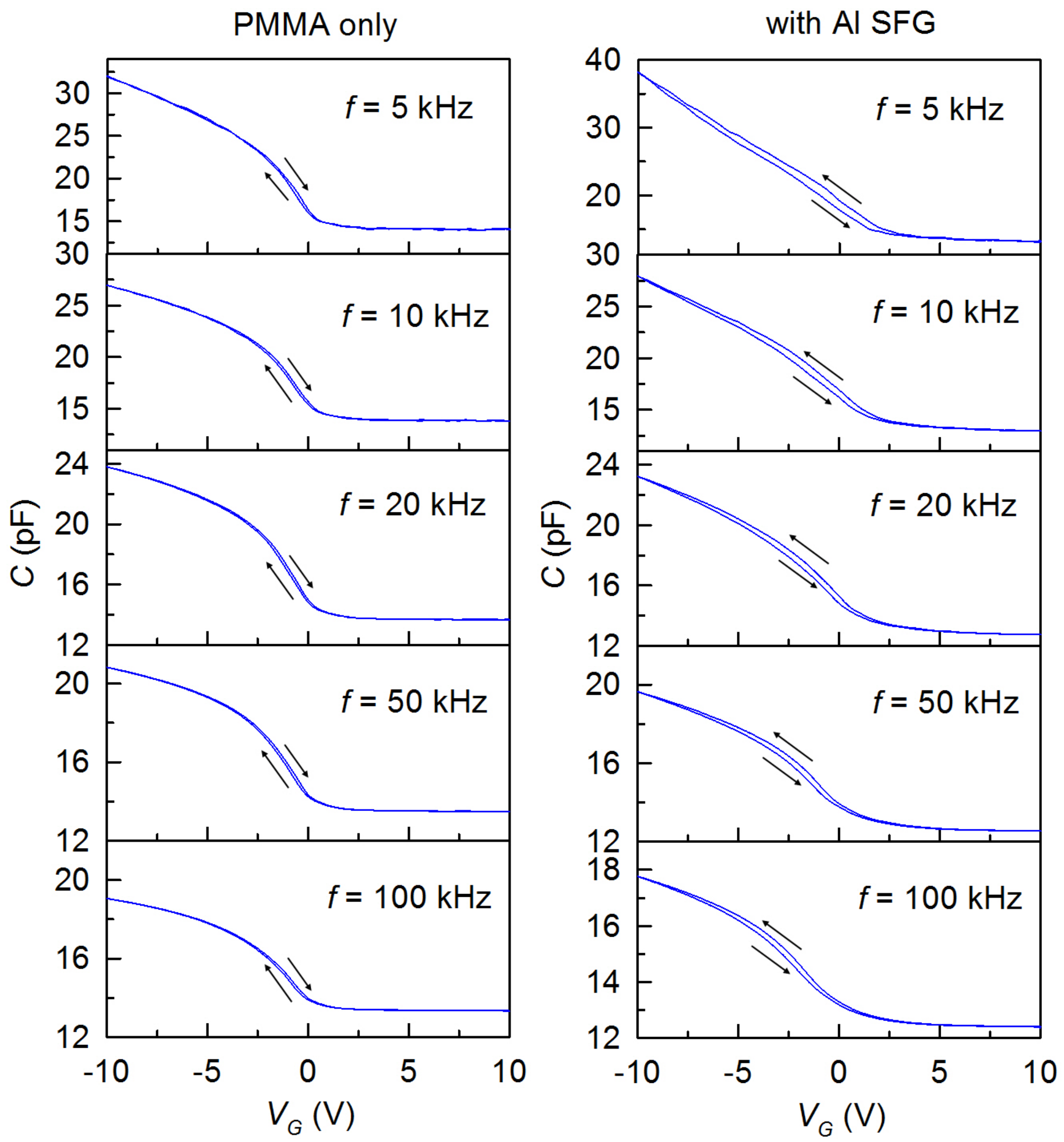

Figure S8. Dual-sweep C-V_G data with different small-signal frequencies, measured on the DNTT OFETs with and without an Al SFG structure. The arrows indicate the direction of voltage sweeps.

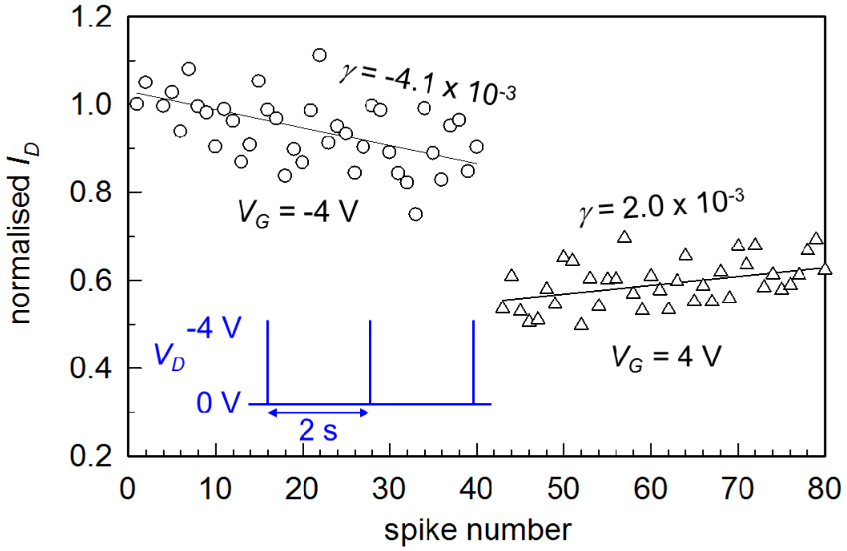

Figure S9. Gate-configurable adaptation behaviour of a DNTT SFG-OFET. Here, V_D spikes of -4 V were applied to the channel at a frequency of 0.5 Hz, while V_G independently changed from -4 V to 4 V in the middle of the test duration. The parameter γ corresponds to the slope of the linear regressions drawn as solid lines.
